# Long-read sequencing unmasks a cryptic three-way translocation resulting in an *ETV6*::*PDGFRB* fusion

**DOI:** 10.1186/s13039-025-00730-7

**Published:** 2025-10-21

**Authors:** Joseph Tripodi, Douglas Tremblay, Daiva Ahire, Vesna Najfeld

**Affiliations:** 1 Department of Pathology and Molecular Based Medicine, Icahn School of Medicine at Mount Sinai, New York, NY 10029 USA; 2 Division of Hematology/Oncology, Department of Medicine, Tisch Cancer Institute, Icahn School of Medicine at Mount Sinai, New York, NY 10029 USA

**Keywords:** Long-read sequencing, *PDGFRB*, Cryptic translocation, Myeloid/lymphoid neoplasms, Tyrosine kinase inhibitors, *ETV6*::*PDGFRB*, Genomic complexity, Precision oncology

## Abstract

**Background:**

Myeloid/Lymphoid Neoplasms (MLN) with eosinophilia and *PDGFRB* rearrangements are rare but distinct hematologic malignancies driven by the constitutive activation of the *PDGFRB* tyrosine kinase through gene fusions. These neoplasms are sensitive to tyrosine kinase inhibitors (TKIs) such as imatinib, which often leads to rapid and durable molecular remissions. However, diagnostic challenges frequently arise from cryptic rearrangements, necessitating comprehensive molecular approaches.

**Case presentation:**

A 37-year-old male patient initially presented with pancytopenia and a splenic infarct; subsequent bone marrow findings were suggestive of a myeloid/lymphoid neoplasm. Initial conventional cytogenetic analysis and fluorescence in situ hybridization (FISH) identified a *PDGFRB* gene rearrangement but were unable to fully resolve the structural complexity of the underlying genomic alteration. Long-read sequencing helped resolve a complex three-way translocation involving chromosomes 5, 12, and 20, precisely defining the *ETV6::PDGFRB* fusion with base pair resolution, and identified the partner gene (*KAT14*) on chromosome 20p. Following the diagnosis, the patient was started on imatinib therapy and has since achieved clinical and hematological improvement.

**Conclusion:**

This case highlights the significant diagnostic utility of long-read sequencing in uncovering and characterizing cryptic and complex genomic rearrangements that are frequently missed by conventional methods. Accurate molecular characterization is critical for disease classification, guiding targeted therapeutic decisions, and ultimately improving patient outcomes in *PDGFRB*-rearranged neoplasms.

## Background

Myeloid/lymphoid neoplasms (MLN) with eosinophilia and tyrosine kinase gene fusions are hematologic malignancies driven by rearrangements which lead to the kinase domain being constitutively activated, thereby resulting in cell signaling dysregulation that promotes proliferation and survival [1]. These neoplasms are characterized by the expression of fusion genes involving specific receptor tyrosine kinases, primarily *PDGFRA*, *PDGFRB*, or *FGFR1*. MLN with eosinophilia and *PDGFRB* rearrangements involve gene fusions with up to 40 known partner genes, with the most common of these rearrangements being *ETV6::PDGFRB* fusion resulting from a t(5;12)(q32;p13) translocation [2–4]. The ETV6*::*PDGFRB oncoprotein leads to ligand-independent dimerization and activation of the PDGFRB kinase domain, promoting uncontrolled cell proliferation and survival through downstream signaling pathways such as RAS/MAPK and PI3K/AKT [5]. Clinically, these neoplasms present frequently with marked peripheral eosinophilia and may manifest with myeloproliferative features, lymphoid involvement, and/or progression to acute leukemia. Unlike other eosinophilic disorders, *PDGFRB*-rearranged neoplasms are highly sensitive to tyrosine kinase inhibitors (TKIs) such as imatinib, often achieving rapid and durable molecular remissions [6]. However, due to the diversity of fusion partners and breakpoint locations, comprehensive molecular diagnostics—via karyotyping, fluorescence in situ hybridization (FISH), or RNA sequencing—are critical for accurate detection and therapeutic decision-making [7]. We report a case in which conventional cytogenetics and FISH studies detected a *PDGFRB* gene rearrangement, but long-read sequencing revealed its cryptic complexity, uncovering a three-way translocation that included the *KAT14* gene on chromosome 20p as a partner gene. This case underscores the complementary role of long-read sequencing in surpassing the resolution of standard cytogenetic and FISH methods, enabling precise and comprehensive characterization of clinically significant rearrangements.

## Case presentation

In March 2025, a 37-year-old male patient presented for an initial evaluation to our clinic for pancytopenia discovered in the setting of a recent emergency department admission for splenic infarct. Five years prior, he had abnormal pre-operative blood work for knee arthroscopy and was referred to a hematologist but did not follow up. Upon arrival at our emergency department, the patient was experiencing several episodes of abdominal pain and was diagnosed with a splenic infarct with splenomegaly (measuring 15.6 cm in coronal oblique dimension) and hepatomegaly (measuring 16.8 cm in the craniocaudal dimension). Complete blood tests at that time showed a white blood cell count of 21.6 × 10^9^/L with 37.9% neutrophils, 21.0% lymphocytes, 10.9% monocytes, 10.1% myelocytes, 8.4% band cells, 5.0% eosinophils, 3.4% promyelocytes, 1.7% metamyelocytes, and 0.8% basophils. The hemoglobin was 9.5 g/dL and platelet count was 22 × 10^9^/L. Spleen was palpable to 8 cm below the left costal margin. Upon further review of systems, he reported a nearly 20-pound unintentional weight loss over the last 8 months accompanied by early satiety and bloating as well as nightly drenching night sweats. A bone marrow biopsy was performed and demonstrated a hypercellular marrow (> 95%), trilineage hematopoiesis, myeloid hyperplasia with eosinophilia, and hypolobated atypical megakaryocytes. CD34 + blasts involved less than 2% of marrow cellularity and MF-2 + bone marrow fibrosis. A next-generation sequencing panel targeting 433 genes associated with hematologic malignancies was performed and did not identify any pathogenic mutations.

**Fig. 1:**
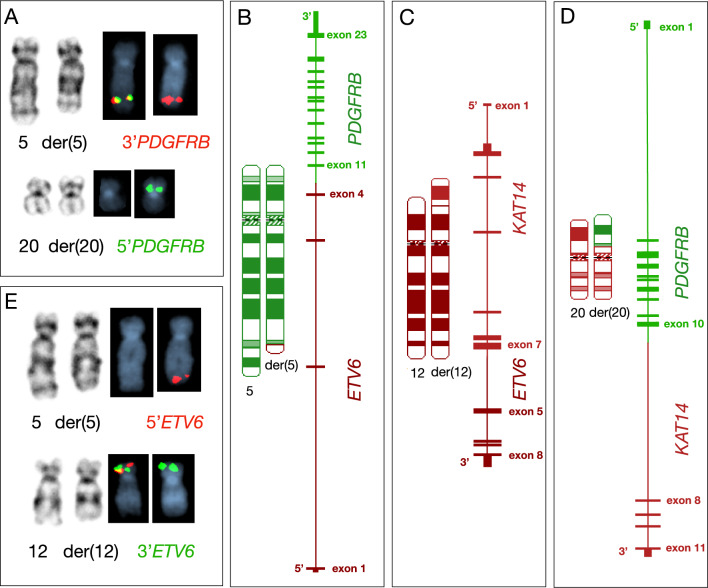
Cytogenetic and Molecular Characterization of a Complex Three-Way Translocation Involving *ETV6*, *PDGFRB*, and *KAT14*. **A** Gene mapping using a *PDGFRB* break-apart FISH probe, with the 3′ region labeled in red and the 5′ region labeled in green, demonstrates a translocation involving chromosomes 5 and 20. **B–D** Chromosome ideograms illustrating a complex three-way translocation resulting in fusion genes: *ETV6::PDGFRB*, *KAT14::ETV6*, and *PDGFRB::KAT14*, located on derivative chromosomes 5, 12, and 20, respectively. **E** Gene mapping using an *ETV6* break-apart probe, with the 5′ region labeled in red and the 3′ region in green, shows a translocation involving chromosomes 5 and 12

Due to a limited bone marrow aspirate as a result of marrow fibrosis, a peripheral blood (PB) sample was submitted for cytogenomic evaluation due to suspicion of a myeloid/lymphoid neoplasm and tyrosine kinase gene fusions. FISH analysis was performed using break-apart probes for *PDGFRA*, *FGFR1*, and *PDGFRB* (Abbott Molecular, Des Plaines, IL, USA) to assess for gene rearrangements. A *PDGFRB* rearrangement was detected in 84% of interphase nuclei, and was reported the following day. Two days later, conventional karyotyping revealed an apparently balanced translocation between chromosomes 5q and 20p, described as 46,XY,t(5;20)(q32;p11.2) in all 20 evaluated metaphases. Metaphase FISH confirmed this finding, demonstrating the 5’ region of *PDGFRB* translocated to the derivative chromosome 20p, while the 3’ region remained on the derivative chromosome 5, consistent with a balanced translocation (Fig. 1A).

To further characterize this rearrangement and identify the partner gene on chromosome 20p, we performed long-read sequencing using Oxford Nanopore technology. Nanopore long-read sequencing works by passing single-stranded DNA or RNA molecules through a nanopore embedded in a membrane. As each nucleotide passes through the pore, changes in the electrical current are measured. These current disruptions are then decoded in real-time to determine the nucleotide sequence, yielding long, continuous reads without the need for prior amplification that span large genomic regions, including breakpoints. Whole-genome sequencing was performed achieving an average read depth of 40x. Structural variant analysis was conducted using Sniffles2, and final results were available within 72 h of sequencing initiation. While FISH helped guide the analysis toward *PDGFRB*, the complex three-way translocation involving chromosomes 5, 12, and 20—affecting the *PDGFRB*, *ETV6*, and *KAT14* genes, respectively—could only be fully resolved by this novel platform. As shown in Fig. 1B-D, long read sequencing identified precise breakpoints within *PDGFRB* intron 10, *ETV6* intron 4, and *KAT14* intron 7. These breakpoints resulted in the formation of an *ETV6::PDGFRB* fusion gene on derivative chromosome 5, a *KAT14::ETV6* fusion on derivative chromosome 12, and a *PDGFRB::KAT14* fusion on derivative chromosome 20. Subsequent metaphase FISH was performed using an *ETV6* break-apart probe (Abbott Molecular, Des Plaines, IL, USA) and showed the 5’ region of *ETV6* on the derivative chromosome 5 and the 3’ region on the derivative chromosome 12, confirming the presence of an *ETV6::PDGFRB* fusion gene resulting from the complex rearrangement (Fig. 1E). The final karyotype was revised as seq[GRCh38] t(5;20;12)(q32;p11.2;p13) NC_000005.10:g.150129216_qterdelins[NC_000012.12:g.11856659_pter] NC_000012.12:g.11856659_pterdelins[NC_000020.11:g. 18165932_per] NC_000020.11:g.18165932_pterdelin[NC_000005.10:g.150129216_qter]. Shortly after diagnosis, he was started on imatinib 400mg daily and has attained clinical and hematological improvement.

## Discussion and conclusion

Recently, Nanopore long-read sequencing (Oxford Nanopore Technologies, Oxford, United Kingdom) has emerged as a powerful tool for comprehensive genomic characterization of hematologic malignancies which enables real-time analysis within a single assay, providing meaningful results in as little as 15 min [8]. Long-read sequencing provides a major advancement in the detection of large chromosomal abnormalities and structural variants (SVs). Unlike short-read platforms, which often struggle to resolve SVs in repetitive or breakpoint-rich regions, long-read technologies generate extended, contiguous reads that span entire variants—including complex rearrangements, large insertions or deletions, and translocations. While base-calling accuracy for single nucleotide variants (SNVs) remains somewhat limited compared to short-read approaches, the ability of long-read sequencing to accurately resolve complex or cryptic structural alterations represents a significant advantage over conventional cytogenetic and sequencing methods. Moreover, long-read sequencing does not rely on prior knowledge of specific fusion events, enabling the unbiased identification of novel gene fusions that may be missed by targeted methods.

Here we report a cryptic *ETV6::PDGFRB* rearrangement that was identified using long-read sequencing. Although initial conventional cytogenetic and FISH analyses suggested a t(5;20) translocation involving *PDGFRB* and a novel partner gene on chromosome 20, it failed to fully resolve the structural complexity of the rearrangement. Long-read sequencing revealed a cryptic three-way translocation—t(5;20;12) leading to an *ETV6*::*PDGFRB* gene rearrangement. To our knowledge, only four other complex chromosomal rearrangements involving *PDGFRB* have been reported in the literature: [1] t(1;5;11), resulting in a *GPIAP1::PDGFRB* fusion; [2] t(4;5;5)(q23;q31;q33), involving a *PRKG2::PDGFRB* fusion; [3] t(1;12;5;12)(p36;p13;q33;q24), generating an *ETV6::PDGFRB* fusion; and [4] t(5;7;7)(q33.2;q32;q11.2), involving *PDGFRB* with an unidentified partner gene [9–11]. None of these cases utilized long-read sequencing; instead, these studies relied on multiple complementary methodologies—such as cytogenetics, FISH, and other molecular techniques—to infer the complexity of the rearrangements. While three of the four cases demonstrated clinical response to imatinib therapy and were initially diagnosed with a chronic myeloid neoplasm, one patient [t(5;7;7)] was diagnosed with AML and was treated with HiDAC consolidation chemotherapy along with imatinib until successful allogeneic stem cell transplant [10].

While conventional cytogenetics and FISH confirmed a *PDGFRB* gene rearrangement—enabling prompt initiation of TKI therapy—the presence of an unusual three-way translocation and unresolved partner chromosome warranted further investigation for several reasons. First, identifying the precise fusion partner can yield important insights into disease biology and potential mechanisms of treatment resistance, particularly when the partner gene is atypical or novel [12, 13]. Second, characterizing complex rearrangements contributes to the broader understanding of structural heterogeneity in *PDGFRB*-rearranged neoplasms. Third, recent studies have highlighted cases of “chromosomal mimicry,” in which chromosome morphology and FISH patterns appear consistent with known structural variants, yet the expected gene-level fusion is absent on molecular analysis [14]. This underscores the importance of definitive molecular confirmation in select cases.

In conclusion, long-read sequencing represents a powerful complementary technology to existing cytogenomic and RNA-based sequencing methods. Its ability to resolve complex structural rearrangements, including cryptic and multi-chromosomal fusions, makes it particularly valuable in genomically challenging cases.

## Data Availability

No datasets were generated or analysed during the current study.
